# Pooled randomised QUARTET trials assessing effectiveness of a single pill for hypertension

**DOI:** 10.1136/openhrt-2025-003843

**Published:** 2025-12-18

**Authors:** Simone Marschner, Mark D Huffman, Desi quintans, Jody Ciolino, Abigail Baldridge, Danielle Lazar, Emily R Atkins, Graham S Hillis, Mark R Nelson, Markus Schlaich, Anthony Rodgers, Clara K Chow

**Affiliations:** 1 Westmead Applied Research Centre, The University of Sydney, Westmead, New South Wales, Australia; 2 Cardiovascular Division and Global Health Center, Washington University in St Louis, St. Louis, Missouri, USA; 3 Department of Preventive Medicine, Feinberg School of Medicine, Northwestern University, Evanston, Illinois, USA; 4 The University of Sydney, Sydney, New South Wales, Australia; 5 Northwestern University Feinberg School of Medicine, Chicago, Illinois, USA; 6 Northwestern University Data Analysis and Coordinating Center, Northwestern University Feinberg School of Medicine, Chicago, Illinois, USA; 7 Department of Preventive Medicine, Northwestern University Feinberg School of Medicine, Chicago, Illinois, USA; 8 Access Community Health Network, Chicago, Illinois, USA; 9 Health Systems Science, The George Institute for Global Health, Newtown, New South Wales, Australia; 10 UNSW, Sydney, New South Wales, Australia; 11 Royal Perth Hospital and Medical School, The University of Western Australia, Perth, Australia; 12 Discipline of General Practice, University of Tasmania Menzies Institute for Medical Research, Hobart, Tasmania, Australia; 13 Dobney Hypertension Centre, The University of Western Australia, Perth, Australia; 14 Royal Perth Hospital, Royal Perth Hospital, Perth, Western Australia, Australia; 15 Cardiovascular, The George Institute for Global Health, Sydney, New South Wales, Australia; 16 Westmead Applied Research Centre, The University of Sydney, Sydney, New South Wales, Australia

**Keywords:** Hypertension, Medication Adherence, Quality of Health Care

## Abstract

**Abstract:**

**Background:**

Hypertension is a major cause of premature death worldwide, controlled by only one in five adults. Two trials (Australia and USA) found a single quadpill containing a quarter dosage of four classes of medication effective in reducing blood pressure (BP) among participants with hypertension. By pooling these trials, we can estimate the overall benefit of the quadpill and its heterogeneity across subgroups and two important barriers for BP control: clinician medication inertia and participant medication adherence.

**Methods:**

In a prespecified pooled individual participant data analysis of two QUARTET randomised, multicentre, double-blinded trials in people with hypertension using ≤1medication, quadpill (irbesartan (37.5 mg) (Australia)/candesartan (2 mg) (USA)), amlodipine (1.25 mg), indapamide (0.625 mg), bisoprolol (2.5 mg) unattended office systolic BP (SBP) at 12 weeks was compared with initial monotherapy (irbesartan (150 mg) (Australia), candesartan (8 mg) (USA)). Heterogeneity was assessed using an interaction term in the mixed cox model. Adherence, ≥80% pill count and treatment inertia were estimated.

**Results:**

In 653 participants (Australia, 591 (91%); USA, 62 (9%)) a significant drop in mean SBP (6.5 mm Hg (95% CI 4.8 to 8.8; p<0·001)) and diastolic BP (5.6 mm Hg (95% CI 4.5 to 6.9; p<0.001)) in favour of the quadpill was found, with less need for uptitration (p<0.001) and less treatment inertia (non-significant: p=0.303). Adherence was high for both treatment arms (over 80%). Compared with monotherapy, the quadpill effect varied by ethnicity (SBP reduced by White (6.9 mm Hg; 95% CI 4.7 to 9.2), Hispanic (3.3 mm Hg; 95% CI 4.0 to 10.6), Asian (12.3 mm Hg; 95% CI 6.2 to 18.5) and Black/other (1.4 mm Hg; 95% CI −9.0 to 6.3), interaction p=0.032).

**Conclusion:**

This prospective individual participant data pooled analysis provides further evidence that the quadpill strategy is superior to initial monotherapy by virtue of improved BP-lowering, less need for uptitration and being associated with less treatment inertia.

**WHAT IS ALREADY KNOWN ON THIS TOPIC**

- There is evidence of the effectiveness of a single quadpill containing a quarter dosage of four classes of medication in reducing blood pressure among participants with hypertension. This promising strategy needs further investigation as a potential solution to global high levels of uncontrolled hypertension.

**WHAT THIS STUDY ADDS**

- This study provides further evidence that the quadpill strategy is superior to initial monotherapy with significantly improved blood pressure-lowering, less need for uptitration and being associated with less treatment inertia.

**HOW THIS STUDY MIGHT AFFECT RESEARCH, PRACTICE OR POLICY**

- This evidence directly contributes to the guidelines supporting the quadpill strategy as a better first line of medication compared with initial monotherapy for patients diagnosed with hypertension.

## Introduction

 Hypertension has significant global impact, affecting an estimated 1.28 billion adults worldwide, causing premature death with a current rate of 21% reaching blood pressure (BP) control.^1^ Two important barriers to BP control are treatment inertia and medication adherence.^2^ Treatment inertia may arise from hesitation in prescribing multiple medications due at least in part to concerns about medication tolerability and adherence, which may be due to resistance in taking many medications. One solution to both these barriers is the low-dose, single pill combination of quarter-standard doses of four common and effective BP medications: the lower dosage addressing tolerability concerns and the single pill addressing participant adherence. The QUARTET trial conducted in Australia found that a single pill containing irbesartan (37.5 mg), amlodipine (1.25 mg), indapamide (0.625 mg) and bisoprolol (2.5 mg) was more effective in lowering BP and maintaining control up to 12 months compared with starting with standard dose monotherapy of irbesartan (150 mg), among participants currently untreated or receiving monotherapy for hypertension.^3^ The QUARTET USA trial suggested a similar direction and magnitude of effect, but with varied levels of significance for systolic BP (SBP) and diastolic BP (DBP).^4^ This study aimed to estimate the pooled effectiveness of the quadpill compared with monotherapy and its heterogeneity across different subgroups in this expanded and diverse cohort, with a secondary aim to estimate the two barriers, namely, treatment inertia and participant medication adherence.

## Method

QUARTET was a multicentre, double-blind, randomised trial conducted as separate trials in Australia and the USA. Eligibility criteria targeted adults (≥18 years) with untreated hypertension defined as a standard observed clinic SBP: 140 mm Hg<SBP<179 mm Hg or 90 mm Hg<DBP<109 mm Hg or both or a daytime average 24 hours ambulatory SBP ≥135 mm Hg or DBP ≥85 mm Hg or both, measured in the last 12 weeks for untreated participants; (2) or a clinical between 130 mm Hg<SBP<179 mm Hg or an 85 mm Hg<DBP<109 mm Hg or both or a daytime average ambulatory SBP ≥125 mm Hg or a DBP ≥80 mm Hg, or both, measured in the last 12 weeks in participants with known hypertension currently treated with one BP-lowering agent. Details of the Australia and US trials are reported in the results papers.^3 4^ While similar in trial design, there were some key differences. The control in the US trial was candesartan (8 mg), while the Australian trial used irbesartan (150 mg) due to manufacturing differences. The US protocol mandated up-titration if BP was not controlled (SBP >130 mm Hg or DBP >80 mm Hg) at 6 weeks, by stipulating that an open label pill of amlodipine (5 mg) supplement the blinded trial drug. The Australian trial had the same recommendation but left this up to the discretion of the local investigator. The US trial recruited from a single federally qualified health network of 35 health centres where most participants have public, limited or no health insurance at all. The Australian trial recruited from ten primary care centres and hospital out participant clinics in four states.

In this pooled individual participant data analysis, the primary objective was to assess effectiveness of the quadpill compared with monotherapy on the average of three unattended SBP measurements at 12 weeks and the heterogeneity of the treatment effect across age, gender, ethnicity, education and whether on baseline monotherapy. A linear mixed model with a random intercept for site and a fixed effect for treatment allocation, visit (6 and 12 weeks), visit-by-treatment interaction and baseline average SBP using individual participant data from both trials. A fixed effect of the interaction of the relevant covariate and the treatment allocation assessed heterogeneity. The secondary outcomes were medication adherence defined as ≥80% use measured by pill count, and treatment inertia, not up-titrating when the participant has uncontrolled BP. The analysis was completed in SAS V.9.4 and R V.4.2.3. R Core Team.^5^

## Results

The Australia (n=591) and US trials (n=62) were balanced across treatment arms.^3 4^ The US cohort, on average, was younger (51.7±11.7 vs 58.6±11.5 years), with higher body mass index (BMI) (33.5±7.3 vs 30.2±5.8 kg/m^2^) and constituted fewer participants with private health insurance (6.0% vs 74.6%). The majority (72.6%) identified as Hispanic (Mexican, Mexican American Chicano, Puerto Rican, Cuban and Other), while the Australian cohort mostly identified as White (81.7%) and Asian ethnicity (Chinese, Japanese, Korean, Vietnam, Cambodia, Laos, the Philippines, Native Malayan, Indonesian, Brunei, India, Pakistan, Sri Lanka, Bangladesh, Iran, Afghanistan) (11.7%) (table 1). Prior to joining the trial, the majority (83.9%) of the US cohort were on monotherapy versus 46.2% of the Australian cohort. The US cohort tended to have less formal education, with lower average income levels than the Australian cohort, given its recruitment strategy.

**Table 1:** Baseline characteristics across the studies and pooled

	Australian n=591	USA n=62	Overall n=653
Baseline SBP	141.3 (13.2)	137.9 (10.7)	141.0 (13.0)
Monotherapy	46.2% (273/591)	83.9% (52/62)	325 (49.8%)
Female	39.8% (235/591)	45.2% (28/62)	40.3% (263/653)
Age (years) mean (SD)	58.6 (11.7)	51.7 (11.5)	58.0 (11.9)
Ethnicity			
White	81.7% (483/591)	8.1% (5/62)	74.7% (488/653)
Hispanic	1.0% (6/591)	72.6% (45/62)	7.8% (51/653)
Asian	11.7% (69/591)	1.6% (1/62)	10.7% (70/653)
Black	0.5% (3/591)	17.7% (11/62)	2.1% (14/653)
Other	5.1% (30/591)	0.0% (0/62)	4.6% (30/653)
Education≤high school	31.5% (186/591)	75.8% (47/62)	35.7% (233/653)
Employment status			
Full-time	48.1% (284/591)	40.3% (25/62)	47.3% (309/653)
Part-time/retired/home duties/student	46.7% (276/591)	29.0% (18/62)	45.0% (294/693)
Unemployed	5.2% (31/591)	30.6% (19/62)	7.7% (50/653)
Income bracket			
$0–$26 k p/a	11.0% (49/445)	69.0% (40/58)	17.7% (89/503)
$26 k–$52 k p/a	15.7% (70/445)	3.4% (2/58)	14.3% (72/503)
$52 k–$130 k p/a	63.4% (282/445)	27.6% (16/58)	59.2% (298/503)
> $130 k p/a	9.9% (44/445)	0.0% (0/58)	8.7% (44/503)
Private health insurance	74.6% (441/591)	12.9% (8/62)	68.8% (449/653)
Marriage or cohabiting	420 (71.1%)/591)	36 (58.1%)/62)	69.8% (456/653)
BMI mean (SD)	30.2 (5.8)	33.5 (7.3)	30.5 (6.0)
Tobacco>once a week			
Never	61.9% (366/591)	67.7% (42/62)	62.5% (408/653)
Former	29.9% (177/591)	12.9% (8/62)	28.3% (185/653)
Current	8.1% (48/591)	19.4% (12/62)	9.2% (60/653)
Alcohol>once a week	63.6% (376/591)	30.6% (19/62)	60.5% (395/653)
Baseline BP treatment	45.7% (270/591)	82.3% (51/62)	49.2% (321/653)
Diabetes diagnosis	7.6% (45/591)	27.4% (17/62)	9.5% (62/653)
Prior CAD	4.4% (26/591)	0.0% (0/62)	24.0% (24/653)
History of depression	22.0% (130/591)	25.8% (16/62)	22.4% (146/653)

SD=standard deviation, BP=blood pressure CAD=Coronary Artery Disease,

BMI, body mass index; BP, blood pressure; CAD, coronary artery disease.

**Figure 1:**
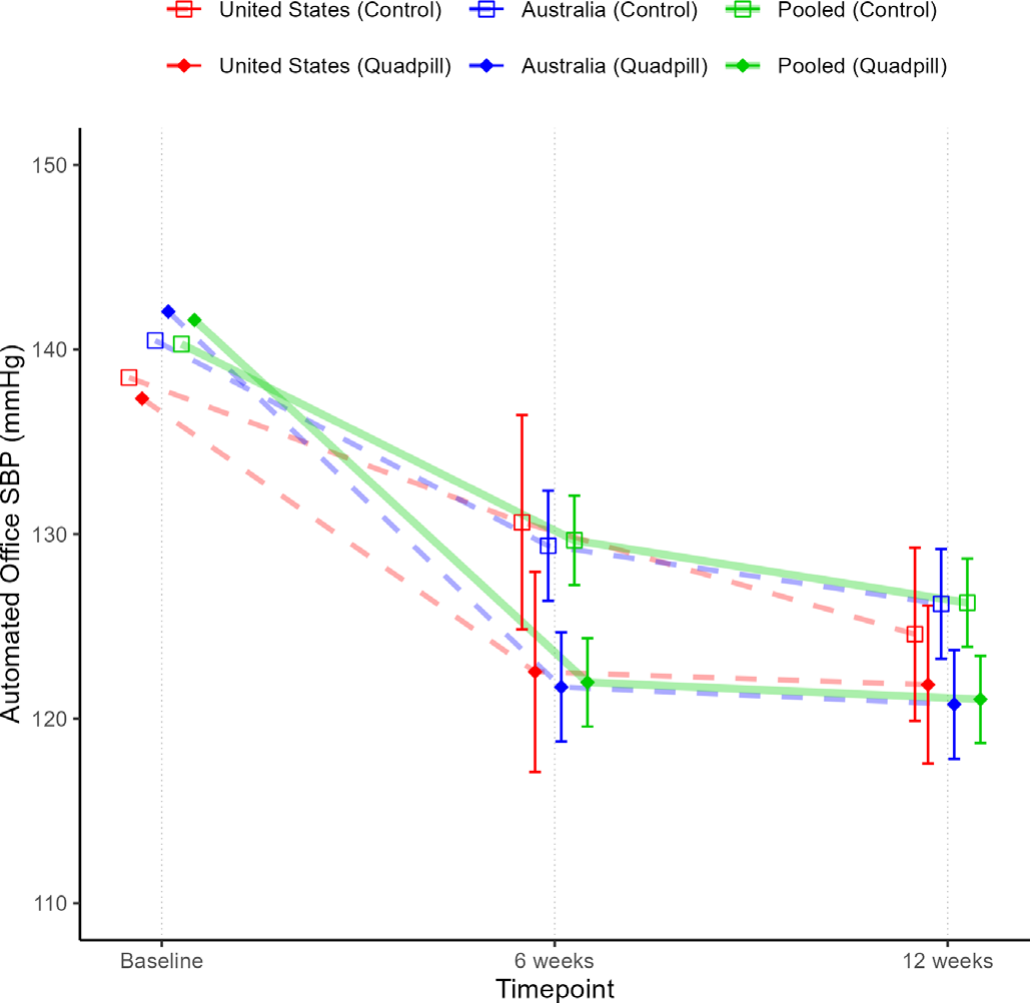
Adjusted automated systolic blood pressure (SBP) across time pooled and by trial.

**Figure 2:**
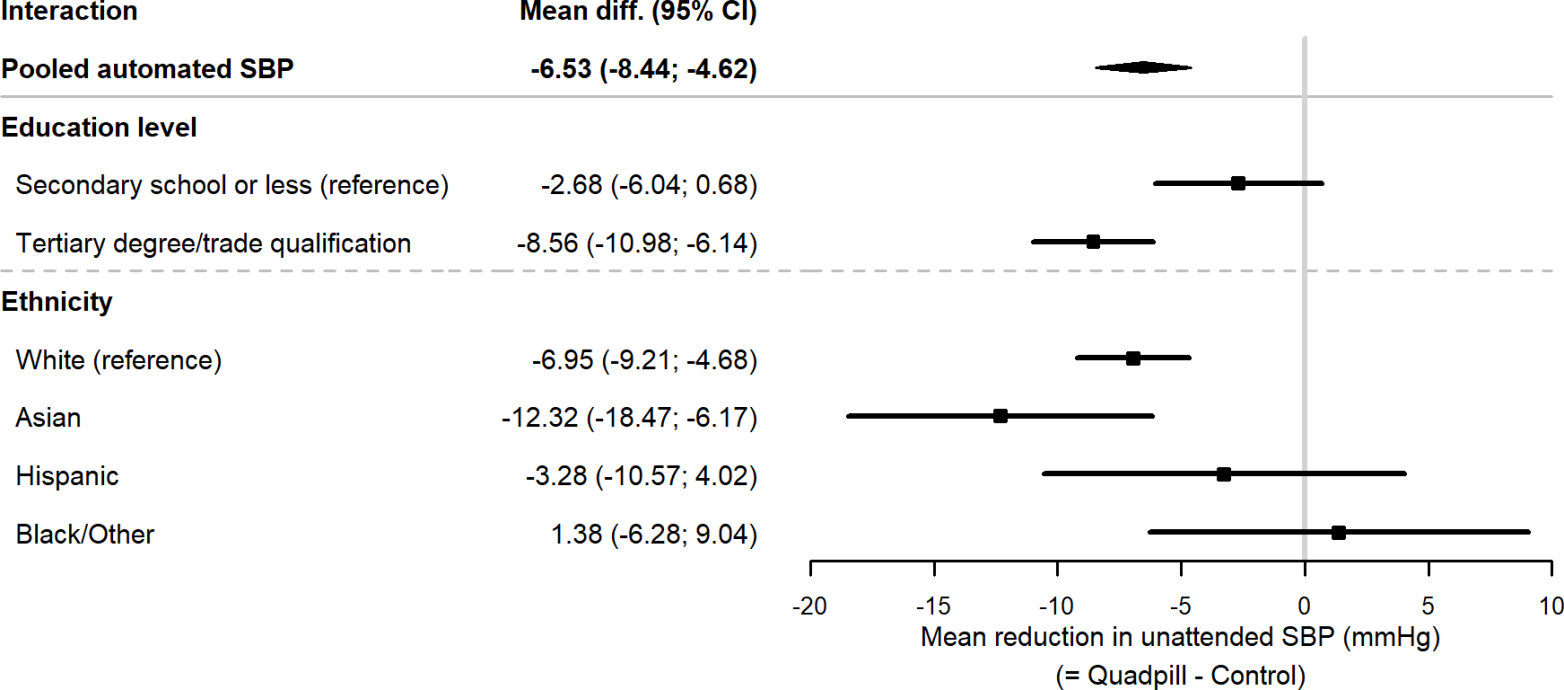
Interaction analysis of automated systolic blood pressure (SBP).

The pooled individual participant data analysis of the primary outcome of unattended office SBP at 12 weeks showed a mean difference between the treatment arms of 6.5 mm Hg (95% CI 4.8 to 8.8; p<0·001) in favour of the quadpill (figure 1). Despite very similar baseline unattended mean office SBP across ethnic groups (White, 141.4±13.3; Hispanic, 137.0±11.6; Asian, 140.5±12.9; Black, 140.1±9.1; and Other, 142.1±11.5 mm Hg), there was a significantly different treatment effect across ethnicities with participants identifying as Asian having the largest drop of 12.3 mm Hg (95% CI 6.2 to 18.5) (figure 2, interaction p value=0.032). Participants with a tertiary degree or trade had a significantly higher drop in SBP than participants with secondary school or less (8.6 mm Hg (95% CI 6.1 to 11.0) vs 2.7 mm Hg (95% CI 0.7 to 6.0), interaction p=0.005). For the remaining subgroups, there was no evidence that the treatment effect on SBP was modified by BMI level, age, gender and whether on baseline monotherapy. There was a significant drop in diastolic BP of 5.6 mm Hg (95% CI 4.3 to 6.9; p<0·001) in favour of the quadpill and no significant difference in serious adverse events with 9 (2.7%) in the treatment arm and 3 (0.9%) in the control arm (p=0.091).

In the pooled analysis, up-titration occurred significantly (p<0.001) more often in the control arm (27.7%; 95% CI 22.8% to 32.6%) compared with the quadpill arm (7.8%; 95% CI 5.2% to 11.0%). Up-titration at 6 weeks was more common in the US trial (35.5%; 95% CI 23.6% to 47.4%), where it was required per protocol, compared with in Australia (15.7%; 95% CI 12.8% to 18.7%), where it was recommended but up to the discretion of the investigator. Treatment inertia at 6 weeks was zero in the USA but 3.0% (18/591) in the Australian trial. In the pooled analysis, treatment inertia was higher in the control arm (control, 11/321 (3.4%); quadpill, 7/332 (2.1%), p=0.303) but not statistically significant. Adherence, at 12 weeks, was similar across the countries at 85.2% (95% CI 82.1% to 88.2%) in Australia and 82.4% (95% CI 71.9% to 92.8%) in the USA and overall by treatment (control, 84.4% (95% CI 80.2% to 88.6%); quadpill, 85.4% (95% CI 81.3% to 89.5%)). Participants tended to exhibit similar adherence levels, regardless of self-identified ethnicity (White, 82.1%; Asian, 80.3%; Hispanic, 75.1%; Other, 77.5%).

## Discussion

The pooled data of the QUARTET trials provided strong evidence of significant and clinically important improvement of SBP after 12 weeks of the quadpill versus monotherapy for participants with untreated hypertension or receiving monotherapy, across a diverse socioeconomic and demographic cohort. There was no significant difference in adherence between the treatment arms, which were high at 12 weeks (over 80%). The US protocol mandated up-titration resulted in over a third of participants being up-titrated, which was significantly higher than the one in five when left to the discretion of the investigator in the Australian trial. Up-titration and treatment inertia were higher in the control arm, but only significant in the former.

The effectiveness of the quadpill varied by ethnicity with the highest effect among the Asian ethnicity, which did not appear to be driven by differences in BMI. Previous literature suggests the association of BP with stroke risk is twice as high for participants of Asian ethnicity compared with those identifying as White,^6 7^ and a randomised trial of the angiotensin-converting enzyme inhibitor perindopril compared with placebo showed larger benefits among those with Asian ethnicity.^8^ These trials have no data on salt intake of participants, but the sodium‐excreting diuretic in the quadpill may provide greater effect for those of Asian ethnicity, who may have higher salt intake and sensitivity.^9^ The recommended drug doses are often lower in Asian countries than in Western countries due to heightened responses to therapeutic medications, including cardiovascular medications,^10^ which may explain the higher effectiveness among the Asian ethnicity participants.

There is evidence that, at 6 months, therapeutic inertia occurred in fewer participants randomised to the quadpill compared with monotherapy.^11^ In this pooled analysis, we found a similar signal at 6 weeks with less treatment inertia in the quadpill arm at 6 weeks. These results highlight the effectiveness of the quadpill in improving the treatment inertia barrier. The increased up-titration and decreased treatment inertia in the US trial, where it was mandated when BP was not controlled, compared with the Australian trial, where it was recommended but up to the physician’s discretion, suggests that direct and explicit guidelines are worthy of consideration^12^ or perhaps simply a reflection on protocol compliance.

Coupled with the finding that trial participants reported positive views regarding the quadpill,^13^ this prospective individual participant data pooled analysis provides further evidence that the quadpill strategy is a better first line of medication compared with initial monotherapy for patients diagnosed with hypertension, with improved efficacy, high adherence and reduced treatment inertia. These types of strategies can help overcome suboptimal hypertension control rates.

## Data Availability

Data are available upon reasonable request.
